# Exercise is required to maintain unacylated ghrelin response in adult male rat skeletal muscle, regardless of dietary fat consumption

**DOI:** 10.1016/j.jpet.2025.103712

**Published:** 2025-09-11

**Authors:** Nicole M. Notaro, Joshua M. Budd, Liam A. Green, Brielle R. Caruso, David J. Dyck

**Affiliations:** Department of Human Health Sciences, University of Guelph, Guelph, ON, Canada

**Keywords:** Unacylated ghrelin, Lipid metabolism, Skeletal muscle metabolism, Hormonal regulation, Exercise, Ghrelin signaling

## Abstract

Unacylated ghrelin (unAG) stimulates fatty acid oxidation (FAO) in isolated male rat skeletal muscle. However, 6 weeks of high-fat feeding results in “ghrelin resistance,” or loss of this effect. Recent work has indicated that sedentary behavior may be a main contributor to the loss of skeletal muscle unAG response, potentially representing an early disruption in lipid metabolism in the development of metabolic disease. Therefore, the objective of this study was to investigate whether exercise is required to maintain the stimulatory effect of unAG on FAO in skeletal muscle and if the exercise intensity needed is dependent on dietary fat intake. Male rats were fed either a low- or high-fat diet for 6 weeks while remaining sedentary, or performing low- or high-intensity exercise. Soleus muscle strips were isolated and assessed for their ability to respond to unAG by increasing FAO. High-intensity exercise preserved unAG response under both low-fat and high-fat dietary conditions, an effect not observed in sedentary or low-exercise groups. Additional soleus muscle strips were collected from all groups to assess the activation of the AMP-activated protein kinase–acetyl-CoA carboxylase axis and Ca2+ signaling in response to unAG; however, these pathways were not found to be significantly activated. Exercise also increased corticotropin-releasing factor 2 receptor content, the putative receptor through which unAG signals in skeletal muscle, whereas high-fat feeding had an overall effect to reduce it. However, unAG treatment did not activate cAMP/protein kinase A signaling. These findings demonstrate a protective role of exercise in maintaining skeletal muscle unAG response, although mechanisms remain to be fully elucidated.

**Significance Statement:**

Unacylated ghrelin stimulates fatty acid oxidation and protects insulin response in skeletal muscle; this response is lost with physical inactivity. We demonstrate that high-intensity exercise preserves this response, potentially due to changes in corticotropin-releasing factor 2 receptor content.

## Graphical abstract

**Figure:**
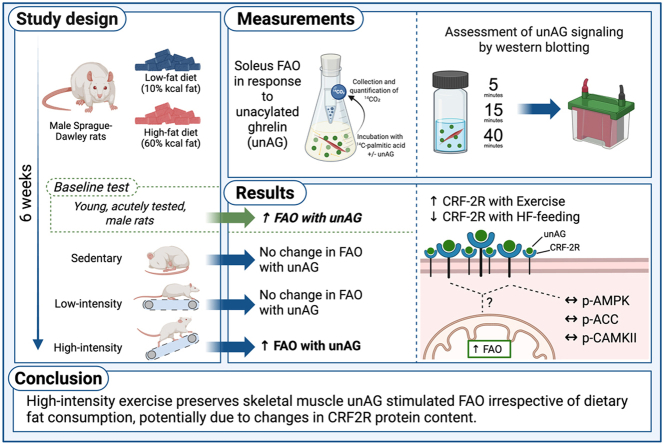

## 1 Introduction

Ghrelin is classically known as an orexigenic hormone, released from the stomach into circulation before a meal to stimulate food intake.^1^, ^2^, ^3^, ^4^ The orexigenic effect of ghrelin is due to its acylated form (AG), which is posttranslationally modified with an octanoic acid, allowing for its interaction with NPY/AgRP neurons of the hypothalamus.^2^^,^^5^^,^^6^ Ghrelin also circulates in an unacylated form (unAG), traditionally considered inactive. However, recent work from our laboratory has demonstrated that both AG and unAG stimulate fatty acid oxidation (FAO) in isolated skeletal muscle from male rats.^7^, ^8^, ^9^, ^10^ Notably, the effect of unAG on FAO in skeletal muscle persists for 2–3 hours after removal of the hormone,^10^ implicating unAG in the metabolism of meal-derived lipids that peak in the circulation ∼2 hours after a meal.^11^ By increasing lipid oxidation, unAG may mitigate the accumulation of reactive intramuscular lipids and prevent insulin resistance, a key factor in the development of type 2 diabetes.

Similar to its response to other hormones that regulate glucose and fat metabolism (eg, insulin, leptin, adiponectin), skeletal muscle becomes less responsive, or resistant, to the effects of unAG after chronic high-fat (HF) feeding.^8^ Implementation of a 4-week high-intensity exercise protocol while HF-feeding maintains skeletal muscle response to unAG.^9^ However, recent work from our laboratory has suggested that skeletal muscle uniquely loses the ability to respond to unAG after 6 weeks of sedentary behavior when consuming a low-fat diet.^9^ Given a potential role of unAG in handling postprandial lipids, ghrelin resistance may represent an early disruption in skeletal muscle metabolism, preventing sufficient oxidation of lipids, leading to their accumulation and subsequent disturbances to insulin signaling.

Importantly, the signaling pathway(s) that facilitate the ability of unAG to stimulate FAO, as well as how they are affected by various diet and exercise conditions, remain relatively unknown. In C2C12 cells, antagonism of the corticotropin-releasing factor 2 receptor (CRF2R) abolishes the stimulatory effect of unAG on glucose uptake.^12^ We have previously found CRF2R receptor content to be reduced in skeletal muscle of HF-fed sedentary animals, and restored with exercise,^8^^,^^9^ corresponding to the ability of unAG to stimulate FAO. However, the effect of exercise training on CRF2R content has yet to be assessed independently of HF-feeding. Additionally, unAG has been shown to activate the AMP-activated protein kinase–acetyl-CoA carboxylase axis (AMPK-ACC) axis,^7^^,^^10^ albeit inconsistently,^13^^,^^14^ with this being lost after 6 weeks of HF-feeding.^8^ Whether this signaling event can be restored with exercise is unknown. Finally, although Ca^2+^ signaling has been independently associated with both CRF2R and ghrelin signaling in other tissues,^13^^,^^15^, ^16^, ^17^ it remains largely uninvestigated in response to unAG in skeletal muscle.

The primary objective of this study was to determine whether regular exercise is required to maintain skeletal muscle response to the FAO-stimulating effects of unAG in male rats, and whether the intensity of exercise that is sufficient to maintain response to unAG is dependent on dietary fat intake. We hypothesized that only animals receiving an exercise intervention would increase muscle FAO in response to unAG, and that low-intensity exercise would be sufficient to maintain this response in low-fat (LF)-fed, but not HF-fed animals. A secondary aim was to examine the activation of various signaling pathways potentially involved in unAG signaling. We hypothesized that unAG-stimulated FAO would correspond to activation of the AMPK-ACC axis and Ca^2+^ signaling proteins.

## 2 Methods

### 2.1 Materials and reagents

Reagents, molecular weight markers, and nitrocellulose membranes for western blots were purchased from BioRad Laboratories. Invitrogen NP40 cell lysis buffer (Cat. No. FNN0021), RestorePLUS Western Blot Stripping Buffer (Cat. No. 46430), Pierce enhanced chemiluminescence Western Blotting Substrate (Cat. No. 32106), and all BCA assay supplies were purchased from Thermo Fisher Scientific. Phenylmethylsulfonyl fluoride (Cat. No. P7626) and protease inhibitor cocktail (Cat. No. P8340) were purchased from Millipore Sigma. The following antibodies were purchased from Cell Signaling Technology: alpha-tubulin (Cat. No. 2144), phospho-AMPK Thr172 (Cat. No. 2531), AMPK (Cat. No. 2532), phospho-calcium/calmodulin-dependent protein kinase II (CAMKII) Thr287 (Cat. No. 12716), CAMKII (Cat. No. 4436), phospho-ACC Ser79 (Cat. No. 11818), ACC (Cat. No. 3676), phospho-(Ser/Thr) protein kinase A (PKA) Substrate (Cat. No. 9621), Phospho-CREB Ser133 (Cat. No. 9198), and CREB (Cat. No. 4820). Antibodies for cytochrome c oxidase (Cat. No. ab16056), citrate synthase (CS; Cat. No. ab129095), and CRF2R (Cat. No. ab104368) were purchased from Abcam. Goat anti-rabbit secondary antibody (Cat. No. 1706515) was purchased from BioRad Laboratories. UnAG (Cat. No. 4049310) was purchased from BaChem (Torrence). Antisauvagine-30 (Cat. No. 34271) was purchased from Cayman Chemical. D-glucose (Cat. No. G8270), sodium bicarbonate (Cat. No. S5761), Dulbecco’s Modified Eagle’s Medium (Cat. No. D5030), bovine serum albumin (Cat. No. A7030), palmitic acid (Cat. No. P0500), caffeine powder (Cat, No. C0750), and benzethonium hydroxide (Cat. No. B2156) were purchased from Millipore Sigma. ^14^C-palmitic acid (Cat. No. ARC172A) was purchased from American Radiolabeled Chemicals Inc.

### 2.2 Animals

All procedures in this study were approved by the Animal Care Committee at the University of Guelph and were in accordance with the Canadian Council on Animal Care Guidelines. For all experiments, male Sprague-Dawley rats were obtained from Charles River Laboratories Canada. Rats were housed in groups of 2 to 4 per cage under a 12h:12h reverse light-dark cycle for the duration of the experiments with ad libitum access to food and water. Upon arrival, rats were given a standard laboratory rodent diet (Teklad 2914 rodent diet, Envigo) and allowed to acclimate for 1 week before beginning any experimental procedures. For initial quality control and cAMP/PKA signaling experiments, rats were obtained at approximately 6 weeks of age (150–175 g). For all dietary intervention and exercise-training experiments, rats were obtained at approximately 5 weeks of age (101–125 g) and allowed to acclimate for 1 week before beginning 6 weeks of ad libitum feeding on either a HF (60% kcals fat; Research Diets D12492) or a sucrose-matched LF (10% kcals fat; Research Diets D12450J) diet. Body weight was measured weekly. Food intake was measured weekly in grams consumed per cage and divided by the number of animals in that cage. Caloric intake was calculated as the amount of food consumed (g) on average per animal within each cage, multiplied by the energy content of the diet (3.85 kcal/g for LF diet, 5.24 kcal/g for HF diet).

### 2.3 Exercise training

After an initial 2 weeks on their diets, a subset of both LF-fed and HF-fed animals began either a low (LoEx)- or high (HiEx)-intensity treadmill training protocol (Treadmill Cat. No. 130254, Columbus Instruments), or remained sedentary (SED). Both exercise protocols consisted of 1 hour of treadmill exercise, 5 days/week for the remaining 4 weeks of the study duration. Rats were exercised at a consistent time each day in their dark (active) cycle. In the week before beginning the exercise training, all rats were familiarized with the treadmill in periods lasting less than 10 minutes. The low-intensity exercise was conducted at 8 m/min at a 0% incline for the 4 weeks. The high-intensity treadmill protocol began at 10 m/min at a 0% incline, increasing each day and reaching 15 m/minute by the end of the first week. The second week, the incline was set to 5% and the speed was increased each day to reach 20 m/min. The third week, the treadmill was set to a 10% incline and 25 m/minute. This speed was held constant in the 4th week, with the incline further increased to 15%. Additionally, in the last week, rats were subjected to 6, 1-minute sprints at 35 to 40 m/min at a 10% incline after their regular training. This is similar to exercise protocols used previously in our laboratory.^9^^,^^18^^,^^19^ Rats were not exercised for the 48 hours before tissue collection and were fasted overnight.

### 2.4 Intraperitoneal glucose tolerance testing

Intraperitoneal glucose tolerance testing (IPGTT) was conducted at the end of the 6th week of the diet period (4th week of training). Before glucose tolerance testing, rats were withheld from treadmill training for 48 hours and fasted overnight. All blood glucose measurements were made using the FreeStyle Libre flash glucose monitoring system with FreeStyle Precision blood glucose test strips (Abbott Diabetes Care Inc). To obtain a baseline blood glucose measurement (t = 0), a small cut was made in the end of the tail, and blood was collected from the tail vein. This baseline measurement was used as the fasting blood glucose (FBG) value. A glucose bolus (20% solution) was administered with an intraperitoneal injection at a dose of 2.0 g/kg animal body weight. Subsequent blood glucose measurements were made at 15, 30, 45, 60, 90, and 120 minutes. Area under the curve (AUC) was calculated as the total peak area above baseline.

### 2.5 Palmitate oxidation measurements in isolated soleus muscle incubations

The morning of tissue collection, rats were re-fed for ∼30 to 40 minutes at the beginning of their dark cycle. Food was then removed for the subsequent 2 hours before beginning experimental procedures. This feeding protocol has been previously shown by our laboratory to reduce circulating insulin and ghrelin at the time of collection,^7^^,^^20^ removing endogenous levels of these hormones as confounding factors. Rats were then anesthetized via an intraperitoneal injection of sodium pentobarbital (6 mg/100 g b.w.) before tissue collection. After tissues were removed, animals were sacrificed by injection of a sodium pentobarbital bolus to the heart. Soleus muscle strips weighing 20 to 35 mg were excised tendon to tendon and placed in vials containing a Dulbecco’s Modified Eagle’s Medium incubation media supplemented with 4% bovine serum albumin, sodium bicarbonate, 5mM glucose, and 1 mM palmitate that had been gassed with 95% O_2_ 5% CO_2_. Muscle strips were allowed to equilibrate in this buffer for 45 minutes at 30 °C in a shaking water bath before being transferred to a similar incubation buffer containing ∼1 *μ*Ci of radiolabeled ^14^C palmitic acid with or without 150 ng/mL unAG. Strips were incubated in this vial sealed with a rubber stopper for 1 hour and 15 min. At the end of the incubation, benzethonium hydroxide was delivered via injection into an Eppendorf tube suspended in the vial from the rubber stopper. The buffer medium and muscle were then acidified by the addition of 1.5 mL of 1M sulfuric acid, and the liberated CO_2_ was collected in a shaking water bath for an additional 2 hours. The Eppendorf tube containing the benzethonium hydroxide and trapped ^14^CO_2_ was then carefully removed and placed into a scintillation vial containing 5 mL of CytoScint scintillation cocktail (Cat. No. 882452, MP Biomedicals). Tendon weights were subtracted from initial muscle weights for normalization of FAO. After a minimum of 24 hours of quenching in complete darkness, liquid scintillation counting was performed for 5 minutes per sample using a PerkinElmer Tri-Carb LSC 4910 TR liquid scintillation counter. We also performed an initial set of quality control incubations to confirm that unAG-stimulated FAO in soleus muscle from young male rats. In these initial experiments, we included a 5 mM caffeine condition to serve as a positive control.

### 2.6 Signaling experiments

In a separate set of soleus muscle strips, protein signaling was assessed under similar incubation conditions to those used to measure palmitate oxidation, except that radiolabeled palmitic acid was not present. Muscle strips were incubated with or without 150 ng/mL unacylated ghrelin for 5, 15, or 40 minutes. Muscle strips were blotted, frozen in liquid nitrogen, and stored at −70 °C. CAMKII phosphorylation was assessed at 5 minutes, AMPK phosphorylation was assessed at 15 and 40 minutes, and ACC was assessed at 40 minutes. COXIV, CS, and CRF2R protein expression were also assessed in the 15-minute untreated soleus tissue. As shown under Results, we did not detect significant stimulation of AMPK, ACC, or CAMKII by unAG. We therefore subsequently tested for changes in PKA and cAMP signaling in an additional set of animals. Given our current results demonstrating a potential role for CRF2R, we performed this set of signaling experiments in the presence of the CRF2R selective antagonist. For cAMP/PKA signaling experiments, muscle soleus strips were incubated for 20 minutes with or without 150 ng/mL of unAG, with an additional condition containing unAG + 1.0 *μ*M antisauvagine-30. This dosage was based on previous work.^12^^,^^21^ In antisauvagine-30 conditions, muscle strips were preincubated with antisauvagine-30 for 75 minutes. Antisauvagine-30 was also present during the 30-minute incubation.

### 2.7 Western blotting

Frozen muscle tissue was chipped into 20 to 30 mg pieces and placed into homogenizing tubes with 2 lysis beads containing ice-cold cell lysis buffer (containing Na_3_VO_4_ and NaF to inhibit serine/threonine and tyrosine phosphatases, respectively) supplemented with a protease inhibitor and phenylmethylsulfonyl fluoride. Tissues were homogenized using the Qiagen TissueLyser LT (cat. No. 85600) for 2 minutes× 4 with samples resting on ice between each round. Samples were then centrifuged at 4 °C at 1800 g for 15 minutes, and the supernatant was removed. Homogenate supernatant was then subjected to a bicinchoninic acid protein quantification assay and prepared with 2× Laemmli sample buffer to a final concentration of 1 mg/mL. Ten micrograms of protein was loaded on 10% acrylamide gels for the detection of CS, AMPK at 15 min, CRF2R, pPKA substrate, and CREB. Ten micrograms of protein was loaded onto 12.5% acrylamide gels for the detection of COXIV. Twenty micrograms of protein was loaded on 10% acrylamide gels for the detection of CAMKII. Protein (7.5 *μ*g) was loaded onto 7.5% gels for AMPK and ACC at 40 minutes. Gels were then subjected to electrophoresis to separate proteins by molecular weight. Protein was then wet transferred onto nitrocellulose membranes for 1 hour and 15 minutes at 4 °C at 100V. Membranes were then blocked with 5% non-fat skim milk + Tris-buffered saline with Tween 20 (TBST) for 1 hour, washed for 10 minutes with 1x TBST, and incubated overnight at 4 °C with primary antibody (1:1000; 1:3000 for COXIV; 1:750 for p-CAMKII) in 5% bovine serum albumin + TBST on a rocker. After the overnight incubation, membranes were washed with 1× TBST for 30 minutes (2× 15 min) and incubated with anti-rabbit secondary antibody (1:2000 dilution in 1% non-fat skim milk + TBST) for 1 hour at room temperature on a rocker. Membranes were washed again for 40 minutes (2× 15 min in 1× TBST, 10 minute in 1× TBS) before imaging. To visualize bands, membranes were exposed to enhanced chemiluminescence for 1 minute. Quantification was performed using Alpha Innotech Software. In the case of phosphorylated proteins, membranes were stripped and reprobed with a total protein antibody. Alpha-tubulin was used as a loading control for all proteins. For analysis of signaling proteins in dietary intervention/exercise-training experiments (AMPK, ACC, CAMKII), the same control sample was included on each gel to allow for normalization across gels. Protein content levels for experimental samples were quantified relative to the control sample band within each gel.

### 2.8 Statistical analysis

All data are expressed as mean ± standard deviation. Statistical significance was accepted as *P* < .05. All statistical analyses were conducted using GraphPad Prism 9. Palmitate oxidation data from the initial quality control experiment and cAMP/PKA signaling data were analyzed using a repeated measures one-way ANOVA, followed by a Tukey’s post-hoc test if significance was found with the ANOVA. Changes in body weight, FBG, glucose AUCs, mitochondrial protein markers (COXIV, CS), and CRF2R protein content data were analyzed using a two-way (diet × exercise) ANOVA. If significance was found using the ANOVA, data were further analyzed using Tukey’s post-hoc test. Body weight over time and blood glucose concentration over time from the IPGTTs were analyzed within specific timepoints (weeks and minutes, respectively) using a one-way ANOVA followed by a Tukey’s post-hoc test if significance was found at that time point. Food intake and caloric intake over time were analyzed by a repeated-measures two-way (time × diet) ANOVA followed by a Bonferroni post-hoc test. Cumulative food and caloric intake were analyzed using an unpaired t-test. Phospho/total signaling protein content (AMPK, ACC, CAMKII) and palmitate oxidation data were analyzed using a repeated measures three-way (diet × exercise × ghrelin) ANOVA. All datasets were confirmed to be normally distributed using the Shapiro-Wilk normality test. The Robust regression and Outlier removal test (set at 1% maximum false discovery rate) was used to detect and remove any outliers from datasets.

## 3 Results

### 3.1 UnAG stimulates palmitate oxidation in the soleus muscle of young male rats

**Fig. 1:**
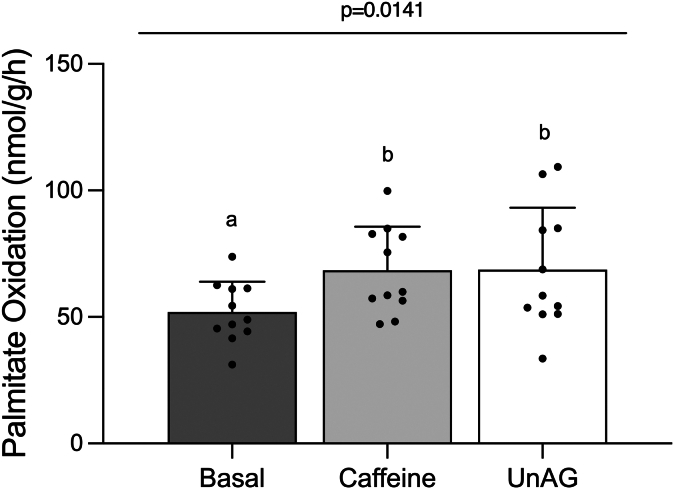
UnAG acutely stimulates palmitate oxidation in the isolated soleus muscle of male rats. Soleus muscle palmitate oxidation in response to 150 ng/mL of unAG or 5 mM caffeine. Data are expressed as mean ± standard deviation. Palmitate oxidation (n = 11/group) was analyzed using a repeated measures one-way ANOVA. *P* < .05 was considered statistically significant. Data sharing a letter are not statistically different. unAG, unacylated ghrelin.

UnAG significantly increased palmitate oxidation in isolated soleus muscle from young male rats (*P* = .0221; Fig. 1). Caffeine, which was used as a positive control, also significantly increased palmitate oxidation compared to untreated muscle (*P* = .0160; Fig. 1).

### 3.2 Effects of HF-feeding and exercise training on body weight and food intake

**Fig. 2:**
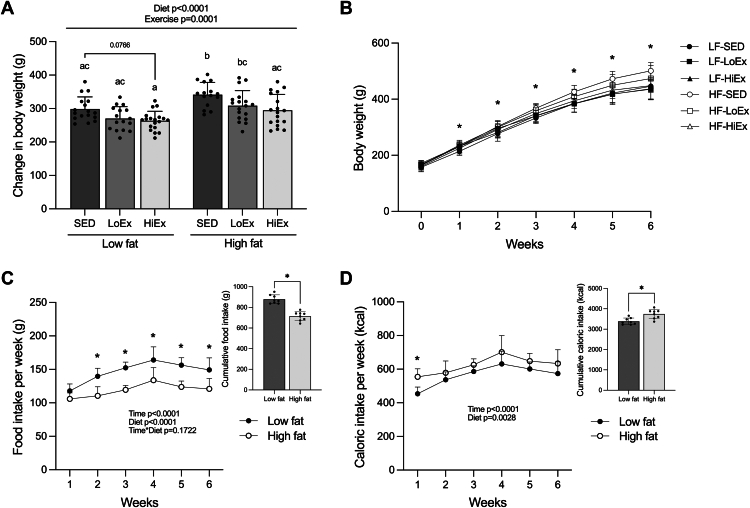
Effect of high-fat feeding and exercise training on body weight and food intake. Change in body weight (A), body weight over time (B), weekly food intake over time and cumulative food intake (C), and weekly caloric intake and cumulative caloric intake (D) following 6 weeks of high-fat feeding with or without 4 weeks of exercise training. Data are expressed as mean ± standard deviation. Changes in body weight (n = 15–19/group) were analyzed using a two-way (diet × exercise) ANOVA followed by a Tukey’s post-hoc test. For body weight over time (n = 14–18/group), significance was determined within specific timepoints using a one-way ANOVA. Food intake over time (n = 8/group) and caloric intake over time (n = 8/group) were analyzed using repeated measures two-way (time × diet) ANOVA followed by a Bonferroni post-hoc test. Cumulative food intake (n = 8/group) and caloric intake (n = 8/group) were analyzed using unpaired t-tests. *P* < .05 was considered statistically significant. Data sharing a letter are not statistically different. ∗Significance in ANOVA between all groups (for change in body weight) or Tukey post-hoc (for food intake and caloric intake) at that time point.

There was a significant main effect of diet (*P* < .0001) and exercise (*P* = .001) on body weight gain over the 6 weeks (Fig. 2A), with differences in weight beginning after the first week of HF-feeding (Fig. 2B). HF-SED animals gained significantly more weight than those in the LF-SED group (*P* = .0266; Fig. 2A). With HiEx, there was a significant reduction in body weight gain in the HF-fed animals (*P* = .0104; Fig. 2A). There was no significant reduction in body weight gain with the LoEx protocol in either diet group when compared to SED animals; however, the change in body weight in the LoEx groups also did not differ from those receiving HiEx (Fig. 2A). LF-fed animals ate significantly more food over the 6 weeks (*P* < .0001; Fig. 2C), however, HF-fed animals had a greater cumulative caloric intake (*P* = .0028; Fig. 2D).

### 3.3 Effect of HF-feeding and exercise training on FBG and glucose tolerance

**Fig. 3:**
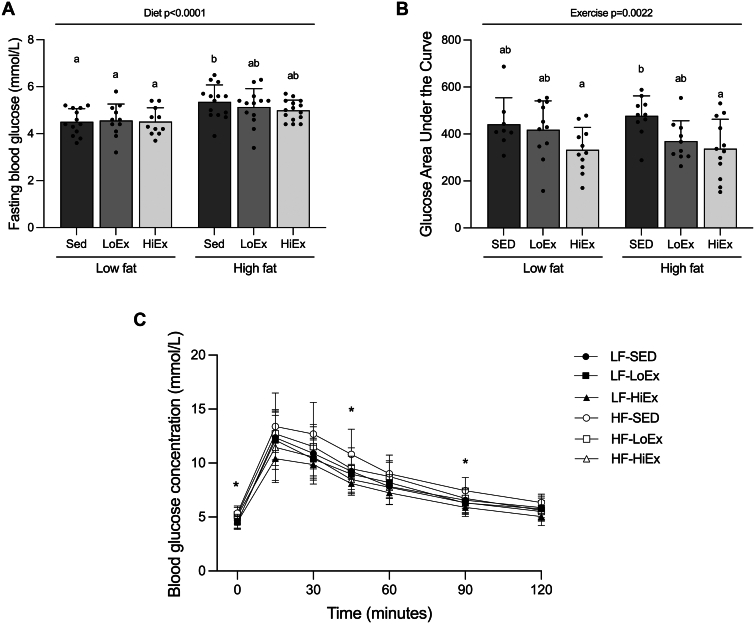
Effect of high-fat feeding and exercise training on glucose tolerance. Fasting blood glucose (A), glucose AUCs (B), and blood glucose concentrations during an IPGTT (C) following 6 weeks of high-fat diet feeding with or without 4 weeks of exercise training. Data are expressed as mean ± standard deviation. Fasting blood glucose (n = 11–15/group) and glucose AUC data (n = 8–12/group) were analyzed using a two-way (diet × exercise) ANOVA followed by a Tukey’s post-hoc test. For blood glucose concentrations during an IPGTT (n = 8–12/group), significance was determined within specific timepoints using a one-way ANOVA followed by a Tukey’s post-hoc test if significance was found. *P* < .05 was considered statistically significant. Data sharing a letter are not statistically different. ∗Significance in ANOVA between all groups at that timepoint. Specific significant differences between groups at each time point are reported in the text.

There was a significant main effect of diet (*P* < .0001), but not exercise (*P* = .6065), on FBG (Fig. 3A). FBG was significantly elevated in the HF-SED animals compared to all LF-fed animals, irrespective of exercise condition (HF-SED vs LF-SED, *P* = .0113; vs LF-LoEx, *P* = .0324; vs LF-HiEx, *P* = .0198; Fig. 3A). Exercise did not reduce FBG in either diet group. During the IPGTTs, blood glucose concentrations were significantly different between groups at 45 (HF-SED vs LF-HiEx, *P* = .0053; HF-SED vs HF-HiEx, *P* = .0209) and 90 minutes (HF-SED vs LF-HiEx, *P* = .0161; Fig. 3C). Overall, there was a main effect of exercise (*P* = .0022), but not diet (*P* = .9048), to lower IPGTT AUC (Fig. 3B). HiEx significantly reduced AUC in HF-fed animals (*P* = .0367); however, there were no differences in the AUC with exercise in the LF-fed animals (Fig. 3B).

### 3.4 Effect of HF-feeding and exercise training on markers of mitochondrial content

**Fig. 4:**
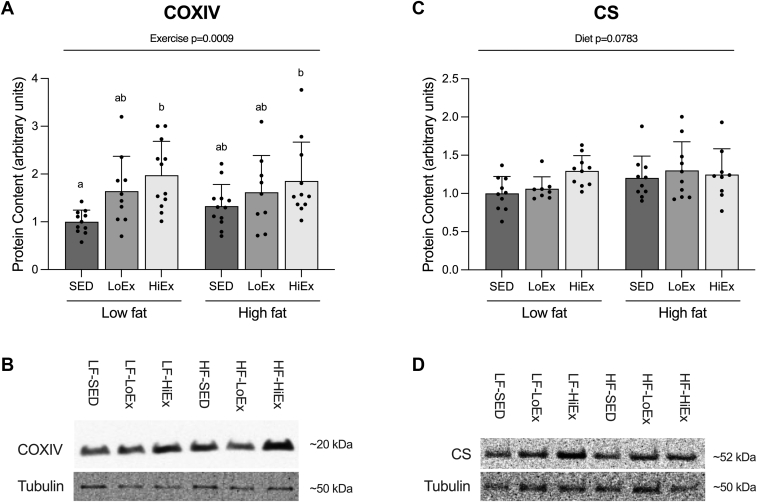
Effect of high-fat feeding and training on markers of mitochondrial content. Cytochrome c oxidase subunit 4 (COXIV; A) and citrate synthase (CS; C) protein content in soleus muscle following 6 weeks of high-fat diet feeding with or without 4 weeks of exercise training. Data are expressed as mean ± standard deviation, and representative blots are shown (Fig. 4, B and D). COXIV (n = 9–12/group) and CS (n = 8–10/group) protein content were analyzed using a two-way (diet × exercise) ANOVA followed by a Tukey’s post-hoc test. *P* < .05 was considered statistically significant. Data sharing a letter are not statistically different.

There was a significant effect of exercise on increasing COXIV protein content (*P* = .0009; Fig. 4A). COXIV protein content was significantly elevated in both the LF- and HF-HiEx animals compared to those in the LF-SED group (LF-SED vs LF-HiEx, *P* = .0069; vs HF-HiEx *P* = .0299; Fig. 4A). There was no effect of diet on CS protein content (*P* = .0783; Fig. 4C). Additionally, for CS, we ran a one-way ANOVA within the diet groups to remove the variance due to diet. This revealed a significant difference between exercise conditions in the LF-fed animals (*P* = .0069), with LF-HiEx having significantly greater CS protein content than both LF-SED (*P* = .0072) and LF-LoEx (*P* = .0494).

### 3.5 Effect of HF-feeding and exercise on unAG-stimulated FAO

**Fig. 5:**
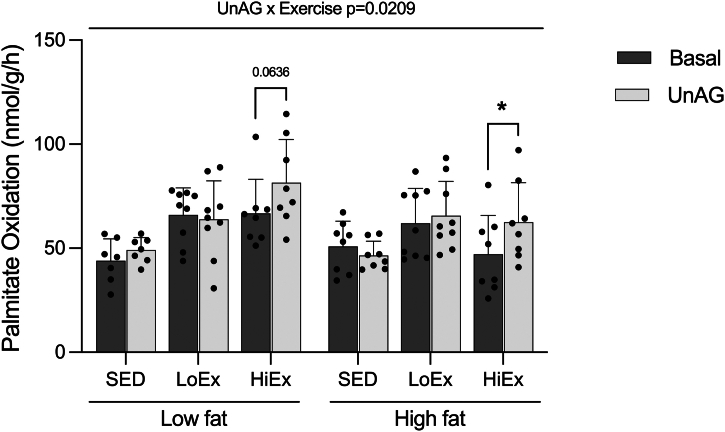
Effect of HF-feeding and training on unAG-stimulated palmitate oxidation. Soleus muscle palmitate oxidation in response to 150 ng/mL of unAG following 6 weeks of high-fat feeding and 4 weeks of exercise training. Data are expressed as mean ± standard deviation. Palmitate oxidation (n = 7–9/group) was analyzed using a repeated measures three-way (diet × exercise × ghrelin) ANOVA. Multiple paired t-tests were used to analyze differences between individual basal-ghrelin pairs. *P* < .05 was considered statistically significant. ∗Significant difference between basal-ghrelin pair.

Previous work from our laboratory has consistently shown unAG to stimulate palmitate oxidation in isolated male rat skeletal muscle.^7^, ^8^, ^9^, ^10^ This effect is lost following 6 weeks of HF-feeding while sedentary,^8^^,^^9^ but is maintained with the implementation of a high-intensity exercise protocol during the HF-feeding.^9^ Interestingly, in this previous study, LF-fed control animals also lost the ability to respond to unAG, suggesting that sedentary behavior is a key factor in the development of ghrelin resistance, irrespective of diet.^9^ In this study, there was an overall interaction effect between unAG and exercise (*P* = .0209; Fig. 5). In the HF condition, unAG significantly increased palmitate oxidation in HiEx animals (*P* = .0439; Fig. 5). There was also a trend toward a significant increase in unAG-stimulated palmitate oxidation in LF-HiEx animals (*P* = .0636; Fig. 5). UnAG did not increase palmitate oxidation in SED or LoEx animals on either diet (Fig. 5).

### 3.6 Effect of HF-feeding and exercise on AMPK-ACC activation in response to unAG

**Fig. 6:**
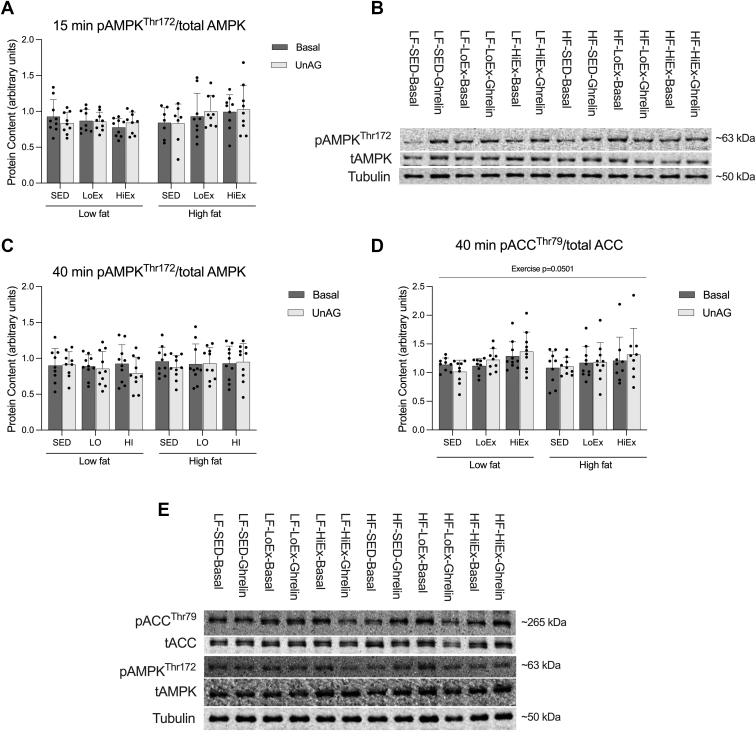
Effect of high-fat feeding and training on phosphorylation of the AMPK-ACC in response to acute unAG exposure. Phosphorylated AMPK protein content in soleus muscle with a 15-minute (A), and AMPK and ACC with a 40-minute (C and D, respectively), exposure to 150 ng/mL of unAG following 6 weeks of HF-feeding and 4 weeks of exercise training. Data are expressed as mean ± standard deviation, and representative blots are shown (B and E). pAMPK/tAMPK at 15 minutes, and pAMPK/tAMPK and pACC/tACC at 40 minutes were analyzed using a repeated measures three-way (diet × exercise × ghrelin) ANOVA. *P* < .05 was considered statistically significant.

Previous work from our laboratory has suggested that unAG may activate AMPK,^10^ as well as the phosphorylation/deactivation of its downstream target ACC.^7^ Additionally, we have shown that this response does not occur after 6 weeks of HF-feeding.^8^ In this study, we did not observe any effect of unAG on AMPK or ACC phosphorylation, regardless of exposure time, or diet/exercise intervention (Fig. 6, A, C, and D).

### 3.7 Effect of HF-feeding and exercise on CAMKII activation in response to unAG

**Fig. 7:**
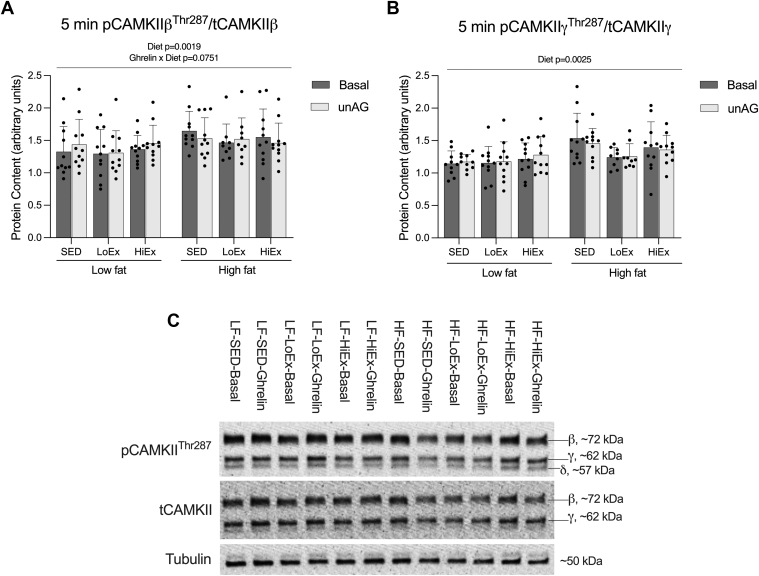
Effect of high-fat feeding and training on phosphorylation of CAMKII in response to 5-minute unAG exposure. Phosphorylated CAMKII*β* and *γ* protein content (A and B, respectively) in soleus muscle with a 5-minute exposure to 150 ng/mL of unAG following 6 weeks of HF-feeding and 4 weeks of exercise training. Data are expressed as mean ± standard deviation, and representative blots are shown (C). pCAMKII*β*/tCAMKII*β* (n = 8–11/group) and pCAMKII*γ*/tCAMKII*γ* (n = 9–11/group) were analyzed using a repeated measures three-way (diet × exercise × ghrelin) ANOVA. *P* < .05 was considered statistically significant.

Our laboratory has previously demonstrated 15 minutes of AG exposure to stimulate CAMKII*γ* phosphorylation at Thr287 in isolated male rat extensor digitorum longus (EDL) muscle; however, this did not occur with unAG, or in either isoform in soleus muscle.^13^ In the present study, there was no significant effect of 5 minutes of unAG exposure and diet on CAMKII*β* phosphorylation at Thr^287^ (*P* = .0751; Fig. 7A). Additionally, there was no effect of 5-minute exposure with unAG on CAMKII*γ* Thr^287^ phosphorylation (Fig. 7B).

### 3.8 Effect of HF-feeding and exercise on CRF2R protein content in soleus muscle

**Fig. 8:**
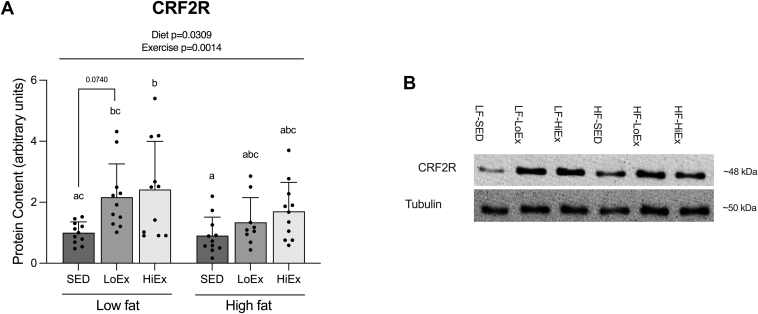
Effect of high-fat feeding and training on CRF2R protein content in soleus muscle. CRF2R protein content in soleus muscle following 6 weeks of high-fat feeding and 4 weeks of exercise training (A). Data are expressed as mean ± standard deviation, and representative blots are shown (B). CRF2R protein content (n = 9−11) was analyzed using a two-way (diet × exercise) ANOVA followed by a Tukey’s post-hoc test. *p* < .05 was considered statistically significant. Data sharing a letter are not statistically different.

Previous findings suggest both ghrelin isoforms act on skeletal muscle through the CRF2R receptor.^12^^,^^21^ Work from our own lab has shown HF-feeding to reduce CRF2R content in soleus muscle,^8^^,^^9^ and that this reduction is restored with exercise training.^9^ Consistent with these findings, there was a main effect of diet to reduce CRF2R protein content (*P* = .0309), with exercise increasing it overall (*P* = .0014; Fig. 8A). Specifically, HiEx increased CRF2R protein content in the LF-fed animals (*P* = .0157; Fig. 8A).

### 3.9 Effect of unAG on cAMP/PKA signaling via CRF2R in soleus muscle

**Fig. 9:**
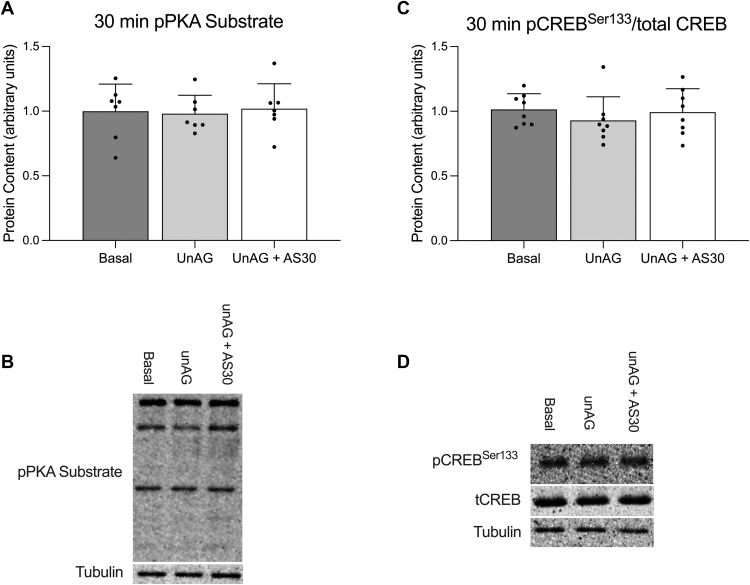
Effect of unAG on cAMP/PKA signaling in soleus muscle. PKA substrate (A) and CREB (C) phosphorylation in soleus muscle following a 30-minute exposure to 150 ng/mL unAG with or without 1.0 *μ*M antisauvagine-30 (AS30). Data are expressed as mean ± standard deviation and representative blots are shown (B and D). pPKA Substrate (n = 7) and pCREB^Ser133^/tCREB (n = 8) were analyzed using a repeated measures one-way ANOVA. *P* < .05 was considered statistically significant.

To further explore the potential role of CRF2R in mediating the effects of unAG in skeletal muscle, additional experiments were conducted to assess cAMP/PKA signaling in response to an acute treatment with unAG, with or without the CRF2R antagonist antisauvagine-30. There was no effect of 30 minutes of unAG exposure on PKA signaling or CREB phosphorylation with unAG, or with the addition of the CRF2R antagonist antisauvagine-30 (Fig. 9, A and B).

## 4 Discussion

### 4.1 Overview

The current study demonstrates that high-intensity exercise preserves the ability of unAG to stimulate FAO in isolated male rat skeletal muscle. Six weeks of sedentary behavior, irrespective of dietary fat consumption, was associated with an inability of unAG to stimulate FAO. However, animals receiving 4 weeks of high-intensity exercise remained responsive. Additionally, in either diet condition, low-intensity exercise was not sufficient to protect against ghrelin resistance. Our findings also reveal that CRF2R protein content, the putative receptor for unAG in skeletal muscle, is reduced with HF-feeding but increased with exercise training. However, this was not associated with cAMP/PKA signaling, a pathway downstream of CRF2R. Finally, we showed that ghrelin resistance and the maintenance of unAG response with exercise are likely not associated with AMPK-ACC or CAMKII activation.

Recent findings from our laboratory have shown that 6 weeks of HF-feeding results in a loss of unAG’s ability to stimulate FAO in skeletal muscle.^8^ Although this previous work found high-intensity exercise during HF-feeding to maintain unAG response, it also suggested that sedentary behavior may be a primary contributor to the development of ghrelin resistance, regardless of diet.^9^ Both HF-feeding^22^, ^23^, ^24^ and sedentary behavior^25^, ^26^, ^27^ are associated with insulin resistance, which is associated with the dysregulation of lipid metabolism. Therefore, given the potential role of unAG in postprandial lipid metabolism, ghrelin resistance may be impairing the oxidation of postprandial lipids, representing an early event in skeletal muscle in the development of metabolic disease. Supporting this hypothesis, our laboratory has demonstrated that unAG can preserve insulin-stimulated glucose uptake in isolated soleus muscle under high palmitate conditions in which acute impairments to insulin signaling typically occur.^8^ This protective effect is lost in the presence of the CPT1 inhibitor etomoxir, indicating this beneficial effect is due to the ability of unAG to increase FAO.^8^ Similarly, in primary rat myoblasts, treatment with AG was found to protect against high palmitate-induced lipid accumulation and impaired insulin action.^28^ Thus, the ability of ghrelin to acutely stimulate muscle FAO is associated with the preservation of normal insulin action.

### 4.2 UnAG signaling and lipid metabolism

#### 4.2.1 CRF2R content may mediate the metabolic effects of unAG in skeletal muscle

AG exerts its central effects through the growth hormone secretagogue receptor (GHSR1a) receptor. Although GHSR1a has been associated with the metabolic effects of AG in some peripheral tissues,^29^^,^^30^ the reported absence of GHSR1a expression in skeletal muscle,^21^^,^^31^ coupled with the metabolic activity of unAG despite an inability to bind GHSR1a, suggests the existence of an alternative receptor. Gershon and Vale first demonstrated that CRF2R may fulfill this role, showing that AG-stimulated glucose uptake in C2C12 cells was abolished by CRF2R antagonism.^21^ Furthermore, treatment of cells with AG and unAG both increased CRF2R mRNA expression levels.^21^ Elbaz and Gershon further explored the role of CRF2R in mediating unAG’s effects, demonstrating that CRF2R antagonism inhibited several effects of unAG in myoblasts, including those related to glucose and lipid metabolism.^12^ In the present study, we found CRF2R content in rat soleus muscle to change in response to various diet and exercise interventions, mirroring the observed loss of unAG response. This is in line with previous findings from our laboratory, which show CRF2R content to decrease with HF-feeding,^8^^,^^9^ and to be restored with high-intensity exercise.^9^

To further explore the role of CRF2R content in mediating the effects of unAG in skeletal muscle, we assessed cAMP/PKA signaling in soleus muscle in response to an acute unAG exposure, with or without the addition of the CRF2R antagonist antisauvagine-30. UnAG treatment did not affect either PKA substrate or CREB phosphorylation. However, it has been previously found that treatment with AG^21^ or unAG^12^ does not independently increase cAMP levels in C2C12 cells. AG and unAG were each only able to increase cAMP levels when treated synergistically with a known CRF2R ligand.^12^^,^^21^

Previous research investigating the role of CRF2R has been restricted to cells.^12^^,^^21^ There are several challenges when attempting to repeat this work in intact mature muscle strips. Inhibitor concentrations may differ, as well as the necessary exposure time. Importantly, we are limited to only a few hours of incubation due to tissue viability, whereas cells have previously been cultured with antisauvagine-30 for up to 48 hours.^12^ Although further investigations are outside the scope of the current work, additional experiments are required to comprehensively determine the role of CRF2R in mediating the effects of unAG in skeletal muscle.

#### 4.2.2 Intracellular signaling pathways associated with unAG in skeletal muscle

Both ghrelin isoforms have been shown to activate AMPK in both cardiac and skeletal muscle.^10^^,^^28^^,^^32^, ^33^, ^34^, ^35^ Specifically, our lab has previously demonstrated unAG to increase AMPK phosphorylation at 15 minutes^10^ and ACC phosphorylation at 30 minutes^7^ in isolated skeletal muscle. In the present study, the phosphorylation of AMPK and ACC did not increase above basal in any of the groups assessed in response to unAG. Thus, either i) exercise was unable to restore the loss of AMPK-ACC signaling seen with HF-feeding/sedentary behavior, even though FAO response was maintained, or ii) stimulation of the AMPK axis is not critical for unAG’s acute effects on FAO.

The role of Ca^2+^ signaling in mediating the central effects of AG,^17^^,^^36^^,^^37^ as well as in peripheral tissues such as the pancreas,^15^^,^^16^ has been demonstrated. However, Ca^2+^ signaling associated with either ghrelin isoform remains understudied in muscle tissue. In isolated ventricular cardiomyocytes, one study,^38^ but not another,^39^ showed AG to increase the amplitude of Ca^2+^ transients. Similar work in skeletal muscle did not find unAG to elicit these same effects.^40^ Work from our laboratory has shown AG to increase CAMKII*γ* Thr^287^ phosphorylation in the glycolytic EDL, but not in the more oxidative soleus.^13^ Additionally, this previous work demonstrated that unAG has no effect on CAMKII*γ* activation in either EDL or soleus muscle.^13^ Although these findings suggest that unAG is not likely to stimulate CAMKII, our previous work may have failed to capture the rapid transience of Ca^2+^ signaling, as CAMKII activation was assessed after 15 minutes of AG/unAG exposure.^13^ Therefore, in the current study, we assessed CAMKII activation at a 5-minute time point. Moreover, we expanded upon previous findings by assessing the activation of multiple isoforms of CAMKII, including *β* and *γ* isoforms. The present study found a trend toward an overall effect of HF-feeding to reduce phosphorylation of CAMKII*β* in response to unAG (Fig. 6). While interesting, this would not explain the changes in unAG-stimulated FAO seen in this study, which appears to be primarily exercise-dependent. However, this may represent an additional level of impairment unique to HF-feeding.

The actual downstream signaling pathways triggered by unAG remain to be elucidated. Although we explored AMPK-ACC and CAMKII activation as potential mediators of unAG, none consistently showed increased activation with unAG. Future work should continue to examine the activation of these signaling pathways with unAG in young rats that exhibit a robust response to unAG.

### 4.3 Development of ghrelin resistance

The mechanism of ghrelin resistance still largely remains unknown. CRF2R reduction may contribute to this resistance, as both this study and previous work^8^^,^^9^ demonstrate its reduction with HF-feeding and increase with high-intensity exercise. However, changes in CRF2R content are not likely to be entirely responsible, as CRF2R protein content in the LF-LoEx animals also tended to increase, yet this group did not have a protected unAG response. The influence of body weight or age on ghrelin resistance cannot be entirely dismissed. In young male rats (∼ 6 weeks of age), skeletal muscle is responsive to unAG, even after 5 days of HF-feeding.^9^ This response in muscle is lost over the next 6 or more weeks, although the exact timeline remains to be established. However, we have previously shown that unAG resistance in adipose tissue develops in as little as 5 days of HF-feeding,^41^ preceding any significant changes in weight or age. To date, we consistently demonstrate unAG to stimulate FAO in skeletal muscle in 2 main groups of male rats: i) young healthy (including previous^7^, ^8^, ^9^, ^10^ and present work) and ii) adult exercise trained (including previous^9^ and present work); these are physiological states associated with muscle growth and differentiation. Emerging evidence suggests a role for unAG in modulating muscle regeneration/growth and function.^40^^,^^42^, ^43^, ^44^, ^45^

### 4.4 Considerations and limitations for ex vivo ghrelin research

Given the ability of ghrelin to stimulate growth hormone release,^46^, ^47^, ^48^ change its acylation status in circulation,^49^^,^^50^ and interact with various serum factors,^51^^,^^52^ ex vivo work has been foundational in elucidating the direct effects of ghrelin isoforms on skeletal muscle. However, when interpreting this work, it is important to consider that using isolated tissue may not reflect what the in vivo effects of ghrelin might be. It has been determined that the acylation status of ghrelin does not remain fixed following its secretion, with AG being deacylated in circulation by an unknown serum factor.^49^^,^^50^ Recent evidence has suggested that unAG can become “reacylated” at the site of tissue signaling.^53^, ^54^, ^55^ This could further complicate unAG research. However, it is important to note that there has been no evidence for ghrelin reacylation to occur at skeletal muscle.

Additionally, while unAG is the predominant circulating ghrelin isoform, it is released together with AG. We have not assessed FAO in response to a combined unAG + AG treatment, although this is more reflective of ghrelin in circulation. Additionally, AG potently stimulates GH release into circulation.^46^, ^47^, ^48^ GH elicits its own independent effects on peripheral tissue metabolism, such as increasing lipolytic sensitivity,^56^ and the induction of acute insulin resistance in both adipose and skeletal muscle.^57^^,^^58^ AG also increases FAO in isolated rat skeletal muscle^7^; however, it remains to be determined whether skeletal muscle loses its response to AG following HF-feeding. Although it is necessary to remove these confounding variables to study the direct effects of unAG on skeletal muscle, they are important to consider when making inferences regarding the role of unAG in vivo.

## 5 Conclusion

We found that high-intensity exercise maintains unAG’s FAO-stimulating effects in male rat skeletal muscle, and that this was associated with changes in CRF2R content. Low-intensity exercise was not sufficient to maintain the muscle’s response to unAG. Given that only HiEx animals were unAG responsive, it is tempting to suggest that there may be a threshold in terms of exercise intensity to preserve unAG-stimulated FAO. Finally, we did not find AMPK, ACC, Ca^2+^, or cAMP/PKA signaling activation to occur with unAG. This work improves our understanding of the disruptions that occur in lipid metabolism following prolonged sedentary behavior, as well as the importance of physical activity in maintaining normal skeletal muscle metabolism.

## Conflict of interest

The authors declare no conflicts of interest.
